# Expanding the Digital, Donor-Assisted Conception Tool to Empower Parental Telling and Talking (TELL Tool) Intervention to the Pregnant and Early Parenthood Periods: Findings From a Qualitative Study

**DOI:** 10.2196/79024

**Published:** 2026-01-13

**Authors:** Patricia E Hershberger, Kirby Adlam, Mary B Richardson, Alison L Miller, Chelsea Fortin, Martha Driessnack, Harold D Grotevant, Susan C Klock, Lauri A Pasch, Agatha M Gallo

**Affiliations:** 1 Department of Health Behavior and Clinical Sciences, School of Nursing, University of Michigan, 400 North Ingalls, Ann Arbor, MI, 48109, United States, 1 734-763-0011; 2 Department of Human Development Nursing Science, College of Nursing, University of Illinois Chicago, Chicago, IL, United States; 3 Department of Health Behavior and Health Equity, School of Public Health, University of Michigan, Ann Arbor, MI, United States; 4 Department of Obstetrics, Gynecology and Reproductive Biology, College of Human Medicine, Michigan State University, Lansing, MI, United States; 5 Center for Reproductive Medicine, Henry Ford Health, Rochester Hills, MI, United States; 6 School of Nursing, Oregon Health and Science University, Portland, OR, United States; 7 Department of Psychological and Brain Sciences, University of Massachusetts Amherst, Amherst, MA, United States; 8 Departments of Obstetrics and Gynecology and Psychiatry and Behavioral Sciences, Feinberg School of Medicine, Northwestern University, Chicago, IL, United States; 9 Department of Psychiatry and Behavioral Sciences, University of California San Francisco, San Francisco, CA, United States

**Keywords:** decision aid, digital health, disclosure, donor conception, embryo donation, family building, family support, gamete donation, parent-child relationship, pregnancy

## Abstract

**Background:**

Many parents who use donor-assisted conception to form their families struggle with telling their children about how they came to be. To address this problem, we created the Tool to Empower Parental Telling and Talking (TELL Tool), a digital, psychoeducational, and decision-support intervention for parents with children aged 1 to 16 years. Recently, we completed a pilot randomized controlled trial of the TELL Tool that showed feasibility, acceptability, and promise. However, in its current version, the TELL Tool does not include content for pregnant, expecting, or new parents with children less than 1 year of age.

**Objective:**

The aim of this formative study was to understand the views of pregnant, expecting, and new parents who used donor-assisted conception to form their families, along with the views of practicing clinicians about disclosure to expand the TELL Tool for use during the pregnant and early parenthood periods in the United States.

**Methods:**

Using a qualitative descriptive approach, a purposive sample of 20 parents and 10 practicing clinicians was recruited using a multifaceted recruitment plan. Each participant completed an in-depth, semistructured interview over Zoom that was recorded, auto-transcribed, checked for accuracy, and subsequently analyzed for themes. The rigorous and accelerated data reduction technique was incorporated into the analytic plan.

**Results:**

Parents comprised pregnant (n=6) or new parents (n=15), as one parent was both pregnant and had a child less than 24 months of age. The 10 clinicians, consisting of an array of multidisciplinary health care professionals, were practicing in fertility/infertility (n=4), obstetrics and women’s health (n=3), and reproductive or family health (n=3) settings. Four themes were identified from the analysis. In “Reasons for What Matters Most,” all parents spoke in favor of disclosing to their children and shared their reasons, while clinicians reported the time limitations in clinical settings. In “Managing Emotions, Conflicts, and Needs,” a myriad of emotions, including conflict, were entwined in the parents’ experiences, and clinicians recognized parents’ feelings and needs as well as their own. Within “Desired Content of a Digital Tool,” participants provided invaluable feedback on what material and content would be helpful to both parents and clinicians. Participants voiced the design features that would resonate or be useful to them or their patients/clients in “Recommended Design and Usability Features.”

**Conclusions:**

Findings illustrate the distinct needs and desires of parents-to-be, new parents, and clinicians about providing expanded content for the TELL Tool that is informed by current evidence and end users, ultimately advancing best practices in this area. Future plans include testing the TELL Tool tailored to this additional developmental period of pregnancy/early parenthood.

## Introduction

Donor-assisted conception, referred to as third-party reproduction, consists of the donation of gametes (sperm, oocytes) and embryos with the specific intent to assist others in establishing a family. As a family-building option, heterosexual couples who cannot conceive with their own gametes, same-sex couples, and single women or single men who want to establish a family often turn to donor-assisted conception. Individuals or couples who are at high genetic risk for passing on an inherited disease to their future children may also opt to use donor-assisted conception to avoid this risk [1,2]. Although prevalence data about donor-assisted conception are challenging to obtain [3-5], recent reports indicate an overall upward trend in the use of donor-assisted conception both globally and in the United States [6-9].

The landscape surrounding parental openness and information sharing (ie, disclosure) to donor-conceived children and individuals has undergone significant changes in the past several decades. Historically, donor-assisted conception was sought and performed in secrecy in efforts to protect parents and their children from psychosocial and legal implications, as the family-building option was viewed as a form of adultery [10,11]. However, in the 1990s, clinicians and researchers began to question the practice of secrecy [12], and a growing number were advocating for openness [13,14]. With the advent of direct-to-consumer genetic tests and the proliferation of social media, clinicians and researchers began to foretell the end of secrecy [15]. Despite this movement, disclosure is not universally accepted worldwide, and some countries mandate anonymity or even ban the use of donated gametes [16,17].

As the number of donor-conceived people (DCP) who are aware of their genetic origins has grown, knowledge about the effects of parents’ openness has been examined. A review of 50 studies with 4666 DCP identified a positive relationship between early parental disclosure of donor conception origins and psychological well-being [18]. Moreover, most DCPs who learned of their conceptual origins in adolescence or adulthood were significantly more likely to be dissatisfied with the timing of the information shared and were more likely to react with negative emotions (eg, shock, confusion, anger) compared with those who were informed earlier [19]. Studies of family functioning in third-party reproduction have substantiated these findings [20-23]. Additionally, the ability of health care professionals to provide health care for children or adolescents is based on the accuracy of their health history [24]. Yet, parents who find it difficult to share information with their children also find it challenging to communicate the donor conception origins to their children’s health care providers [25].

Nationally, US guidelines recommend that individuals and couples undergoing donor-assisted conception receive psychoeducational counseling from a qualified licensed mental health professional who has training and education in third-party reproduction. This counseling is “strongly recommended,” and the guidelines list “disclosure” as a topic for the counseling session. More specifically, the American Society for Reproductive Medicine (ASRM) strongly encourages parents to inform donor-conceived children about the use of donated gametes or embryos in their conception, ultimately leaving the choice to the parents [26]. Individuals and couples undergoing donor-assisted conception can opt to select either a known or “directed” (eg, open-identity/open ID) gamete donors or a “nondirected” (eg, nonidentified, or anonymous) donor, although the ASRM purports that “anonymous” donors have become a rare occurrence due to the increasing access to direct-to-consumer genetic tests [2].

Notably, the European Society of Human Reproduction and Embryology Working Group on Reproductive Donation [27] heightened the need for disclosure and, in 2022, developed “Good Practice Recommendations for Information Provision.” These recommendations state that parents need to be supported and informed on how they can talk age-appropriately with their children about their donor conception origins [27].

To begin to address the issue of disclosure, we created the Tool to Empower Parental Telling and Talking (TELL Tool), a digital, psychoeducational, and decision-support intervention. We designed the TELL Tool to aid parents in making informed and sequential decisions about sharing information (ie, telling and talking) about the donor-assisted conception with their resulting children [28]. The TELL Tool is comprised of 4 modules and includes interactive and multimedia (eg, audio and video clips, visuals, parent workbook) components. To provide age-appropriate information, the tool is tailored to 3 children’s age-based categories (1‐7 years, 8‐12 years, and 13‐16 years) guided by Jean Piaget’s theory of cognitive development about children’s thinking and reasoning [29-31].

As part of the TELL Tool’s development process, we completed a mixed methods alpha test with 9 donor-gamete-recipient parents and 8 clinicians with expertise in counseling parents and families. We then revised and refined the tool [32]. Next, we carried out a randomized parallel, 2-group, attention-controlled clinical pilot trial of the TELL Tool among 75 donor-gamete-recipient parents [33]. Within and across these studies, we found the TELL Tool to be feasible and acceptable to parents, families, and clinicians. Importantly, the TELL Tool also demonstrated promising results in helping parents inform their children about their donor conception origins compared with attention controls [33]. Parents and pediatric nurse practitioners (PNPs) also provided in-depth responses about how to integrate the TELL Tool into pediatric settings [25]. While at present, the TELL Tool is designed for parents who have 1- to 16-year-old children, pregnancy and early parenting periods are critical for the formation of early bonding and attachment relationships between parents and infants and can be a powerful time for intervention [34,35]. To address this gap, the aim of this formative study was to understand the views of pregnant, expecting, and new parents who used donor-assisted conception and the views of practicing clinicians about disclosure to expand the TELL Tool intervention for use during the pregnant and early parenthood periods in the United States.

## Methods

### Study Design

This descriptive, qualitative study was conducted between September 2023 and February 2024 with donor oocyte, sperm, or embryo recipient individuals or couples who were pregnant, expecting, or new parents, as well as health care professionals who were actively practicing in fertility/infertility, obstetrics, women’s health, family health, or reproductive health care settings. The rigorous and accelerated data reduction (RADaR) technique [36] that we [25] and others [37] have used in prior intervention development research informed the qualitative descriptive approach [38,39].

### Ethical Considerations

Institutional Review Board examination and approval of the protocol (#HUM00232409) as exempt were obtained from the University of Michigan prior to the recruitment of participants to ensure the ethical conduct of the study. All participants provided verbal informed consent prior to data collection. The data collected for the study were deidentified to promote participant confidentiality. Each parent and clinician received a $25 gift card for participating in the study.

### Sample and Setting

#### Overview

Parents and clinicians were purposefully selected based on their desired characteristics and the needs of the study [40]. “Parents” were defined as individuals or couples who were pregnant or expecting (eg, partner of pregnant individual), or a new parent with children 24 months or younger. The inclusion of new parents with children equal to or less than 24 months expands the sample to include those who were pregnant or expectant in the recent past. “Clinicians” were defined as health care professionals (eg, physicians, registered nurses, advanced practice nurses, psychologists, social workers) actively practicing and providing health care for individuals and couples who were undergoing or had undergone successful donor-assisted conception.

The inclusion criteria for both parents and clinicians were English-speaking and living in the United States, and the exclusion criteria were non-English speaking and not living in the United States. English-speaking participants were approached because the research team conducting the qualitative interviews was English-speaking. We required that participants live in the United States because governmental policies and cultural norms for donor-assisted conception vary among countries, and the target population for the TELL Tool is parents in the United States. Parent-specific criteria were that the individual or couple were pregnant, expecting, or a new parent through donor-assisted conception, and willing to talk about their views, insights, needs, and experiences about sharing conceptual information with their (future) children during the pregnancy and early parenthood periods. Clinician-specific inclusion criteria were that they provided health care for individuals and couples who were undergoing or had undergone successful gamete donation and were willing and able to express their views about expanding the TELL Tool into the pregnant and early parenthood periods. To ensure the clinician participants were knowledgeable about the TELL Tool, a form that contained key information about the tool, such as its purpose, content, and modules, was provided to each clinician prior to the interview.

The sample size was set at 12‐20 for parent participants and 7‐10 for clinician participants. The sample size and number of interviews were appropriate for achieving the purpose of the study based on our prior research as to when saturation occurred [25,32], International Patient Decision Aid Standards [41-43], expert recommendations for decision aid development [44,45], and experts in qualitative research [46]. The setting for the interviews was web-based, and the participants and the interviewers used a quiet, private location of their choice (eg, a room in their home or a discrete office room).

#### Sample Recruitment

We used a multifaceted recruitment plan that included web-based advertisements posted on websites and other digital media sites that were tailored to pregnant individuals and new parents; flyers were sent to professional email lists that were specific to fertility, obstetrics, or women’s health clinicians; and our research newsletter. The principal investigator or other research team members would also relay information about the study when speaking professionally. All parents meeting eligibility requirements participated in the study; no eligible parent declined participation.

### Data Collection Procedures

The qualitative interviews were conducted by one of two expert qualitative researchers who were also fertility/family (PEH) or obstetrics/women’s health (KA) clinicians, and one junior member of the research team (Ruchi Bhagat). The interviewers completed field notes during and following each interview. Due to the wide geographical distance of the sample, online video/audio conferencing (Zoom Communications, Inc) was used to conduct each interview, which was digitally recorded and transcribed. Of the 20 parents, all used the camera feature, although one turned it off near the end of the interview, and 2 others had it on intermittently; all but 3 of the 10 clinicians used cameras. The research specialists (Ruchi Bhagat and MBR) verified all transcripts for accuracy, corrected errors, and deidentified them to maintain confidentiality.

Two-parent families were encouraged to complete the interviews jointly, to promote understanding of the dynamics of couple interaction. In instances where only one partner in 2-parent families was available to participate, the interview was conducted with only one parent because the TELL Tool intervention was designed to be used by couples, solo parents, or one partner in 2-parent families to allow for a wide range of family types and real-life situations among donor-conceived families. Pilot testing of the semistructured Interview Guide, which contained elements of the think-aloud method for interviewing, occurred during our prior qualitative research with donor-recipient parents and clinicians about disclosure [25,32,47]. The think-aloud method is useful when data are needed to understand how participants think and problem-solve tasks and is recommended for formative intervention research [48-50]. Examples of questions used in the interview guide are provided in Table 1.

**Table 1.** Semistructured interview guide: sample questions.

Question type	Parents	Clinicians
Broad opening question	Think aloud about any thoughts that go through your mind about telling your (future) children about their donor conception origins and say what you are thinking and feeling. Be as detailed or take as much time as you need to express your thoughts and feelings.	Think aloud about any thoughts that go through your mind as you educate and counsel [or how you might educate and counsel] your pregnant or postpartum patients/clients about how they can best prepare for telling their (future) children about their donor conception origins. Be as detailed or take as much time as you need to express your thoughts and experiences.
Follow-up questions	As you think about telling your future children about their donor conception, what matters to you most? Is there anything about your telling your (future) child that you would like to know during the pregnancy or as a new parent? If so, what would that be and why? What materials or features of a digital tool or app would be most beneficial to you during your time as a parent-to-be or new parent? What are some of the challenges (eg, concerns, worries) you have or foresee about telling your (future) child about how they came to be? Are there any other types of information, thoughts, or feelings that are or will be important to you about telling your (future) children that we have not discussed that you would like to share? If so, please tell me what they are.	What features of a digital tool or app do you see as being essential to help pregnant individuals, couples, or new parents achieve disclosure to their (future) children? Prior to having pregnant or new parents use the TELL Tool^a^, what qualifications or features would you like to know about before you recommend it to your patients/clients? What concerns do you think your patients/clients would have about using a digital tool or app such as the TELL Tool? Would you encourage your pregnant or new parents to use a digital tool *like* the TELL Tool? Why or why not? Was there anything else about your patients’ or clients’ experience and the incorporation of a digital tool or app into clinical practice that is important but was not discussed or mentioned? If so, tell me what it was and why it is important.

a TELL Tool: Tool to Empower Parental Telling and Talking.

### Data Analysis

A qualitative descriptive approach guided the data analysis to provide in-depth information required directly from those experiencing the phenomenon under investigation [38]. To complete the analysis, promote trustworthiness, and establish intercoder reliability, 2 participant transcripts were double-coded by 2 researchers, achieving over 80% of coding agreement across each transcript. Disagreements in coding were identified, discussed, and a consensus was established. Transcripts were then coded independently by the 2 researchers who met weekly over 3 months to discuss the coding process and debrief as the themes were developed and identified to capture the views and perceptions of the participants. In instances where dyadic parents participated jointly, each parent’s data was analyzed separately, and couple interaction was examined for shared themes and differences. Consistent with the RADaR technique [36], comprehensive data tables for parents and clinicians were created in Microsoft Word as coding ensued. Then, data reduction and revision iteratively took place to produce more concise data tables that contained the identified themes. As the data tables were merged, reduced, and revised, areas of commonalities and differences across or between participants or the 2 participant groups (ie, parents, clinicians) were identified and grouped, which allowed for data triangulation. Field notes and initial analyses were also reviewed and compared for additional insights and nuances throughout the iterative analytic process, and these data were integrated when appropriate, further supporting intercoder reliability and coding consistency. Last, to further promote trustworthiness through researcher triangulation, an additional member of the research team participated in the analytic meetings, and the larger transdisciplinary research team members provided feedback about the emergent themes. Including multiple team members is a critical component of the RADaR technique [36].

## Results

### Sample Characteristics

The sample consisted of 20 parents and 10 clinicians. Parents were comprised of pregnant (n=6) or new parents (n=15), with one pregnant parent having a child less than 24 months of age. The parents ranged in age from 27 to 52 years, and most identified as female (n=18), White (n=15), and straight (n=18). Among the new parents, their children’s ages ranged from 3 to 24 months. Fifteen parents were either married or living with a partner, and 5 were single (either single by choice or single never married). The donor-assisted conception type used by the parents was oocyte (n=11), sperm (n=5), embryo (n=3), and oocyte and sperm (ie, double donation, n=1). The sample of parents included one straight parent-couple family and one individual who used reciprocal in vitro fertilization (ie, a lesbian couple that uses donated sperm to fertilize one partner’s oocyte to create an embryo, which the other partner carries) and was the gestational parent.

Clinicians (n=10) actively practiced in fertility/infertility (n=4), obstetrics and women’s health (n=3), and family and reproductive health (n=3) settings and consisted of an array of multidisciplinary health care professionals that included Advanced Practice Nurses (n=4), Registered Nurses (n=2), and a Psychologist and Clinical Counselor (n=2). The clinicians reported an average of 16.65 years (SD 15.31) in practice (range 3‐47 y) and nearly all were female (n=8) and White (n=9). Half of the clinicians identified as straight, and the other half represented sexual and gender minorities (ie, lesbian, bisexual, or queer). One clinician also participated as a parent.

### Interviews

The in-depth interviews for the parents ranged from 24 to 75 minutes, with an average of 45 minutes (SD 16 min), while the clinicians’ interviews ranged from 25 to 75 minutes, with an average of 36 minutes (SD 15 min). The one-parent-couple dyad in the sample participated jointly in the interview with their camera on; the one parent-clinician participant simultaneously completed the parent and clinician interviews.

### Themes

#### Overview

Four interrelated themes were identified from the descriptive analysis across the parent and clinician interviews: Reasons for What Matters Most; Managing Emotions, Conflicts, and Needs; Desired Content of a Digital Tool; and Recommended Design and Usability Features. The interrelated themes are not mutually exclusive; rather, they developed as central areas of the participants’ views about disclosure during the pregnant and new parenthood periods. Parent and clinician numeric codes are used in the text below to enhance understanding and provide further insight, yet maintain participant confidentiality. Tables2  3 link the numeric codes to components of the parents’ and clinicians’ (respectively) demographic and contextual information.

**Table 2.** Parent codes linked to demographic components. In certain instances, demographic data components have been disguised to enhance the confidentiality of the participants while maintaining contextual understanding.

Code	Donor anonymity type^a^	Race and ethnicity	Marital status	Donation type	Pregnant, expecting, or new parent^b^
21	Nondirected	White and Non-Hispanic	Married	Egg	Pregnant
23	Nondirected	Asian and Non-Hispanic	Married	Egg	Pregnant
25	Directed	White and Non-Hispanic	Single, never married	Egg, sperm (double donation)	Pregnant
58	Nondirected	Other and Hispanic	Married	Egg	New parent
27	Directed	White and Non-Hispanic	Married	Sperm	Pregnant
42	Nondirected	White and Non-Hispanic	Married	Embryo	New parent
53	Directed	White and Non-Hispanic	Single by choice	Embryo	New parent
29	Nondirected	White and Non-Hispanic	Married	Egg	Pregnant
44	Nondirected	White and Non-Hispanic	Married	Egg	New parent
51	Nondirected	White and Non-Hispanic	Married	Egg	New parent
46	Nondirected	White and Non-Hispanic	Married	Egg	New parent
32	Directed	White and Non-Hispanic	Single, never married	Sperm	New parent
36	Directed	Black African and Non-Hispanic	Single by choice	Sperm	New parent
38	Nondirected	White and Non-Hispanic	Married	Sperm	New parent
34	Nondirected	White and Non-Hispanic	Single by choice	Sperm	New parent
57	Nondirected	White and Non-Hispanic	Married	Egg	New parent
49	Nondirected	White and Non-Hispanic	Married	Egg	New parent
55	Nondirected	Other and Hispanic	Living with a partner	Egg	New parent
40	Nondirected	White and Non-Hispanic	Living with a partner	Egg	New parent
26	Nondirected	Black and Non-Hispanic	Living with a partner	Embryo	Pregnant

a Donor anonymity type at the time of the donation. Nomenclature is based on the recommendations from the Practice Committee of the American Society for Reproductive Medicine & Practice Committee for the Society for Assisted Reproductive Technology [2].

b One participant was pregnant and also a new parent; however, this linkage is not denoted to maintain confidentiality.

**Table 3.** Clinician codes linked to demographic components. In certain instances, demographic data components have been disguised to enhance the confidentiality of the participants while maintaining contextual understanding.

Code	Employment status	Race and ethnicity	Health care discipline	Health care practice setting	Practice experience (years)
504	Full-time	White and Non-Hispanic	Registered Nurse	Fertility/Infertility	4
510	Full-time	White and Non-Hispanic	Registered Nurse	Fertility/Infertility	10
512	Full-time	White and Non-Hispanic	Psychologist	Reproductive/Family Health	12
513	Full-time	White and Non-Hispanic	Advanced Practice Nurse	Fertility/Infertility	13
515	Full-time	White and Non-Hispanic	Social Worker	Fertility/Infertility	22
518	Full-time	White and Non-Hispanic	Advanced Practice Nurse	Obstetrical and Women’s Health	18
533	Full-time	Black and Non-Hispanic	Clinical Counselor^a^	Reproductive/Family Health	3
541	Part-time	White and Non-Hispanic	Advanced Practice Nurse, Midwife	Obstetrical and Women’s Health	41
547	Full-time	White and Non-Hispanic	Physician	Obstetrical and Women’s Health	40
554	Full-time	White and Non-Hispanic	Advanced Practice Nurse, Midwife	Reproductive/Family Health	4

a Bachelor’s degree prepared.

#### Theme 1: Reasons for What Matters Most

Throughout the interviews, parents reflected on the importance of open communication with their (future) children. All parents spoke in favor of disclosing to their children and shared their reasons for it. Fifteen of the participants explicitly mentioned “honest,” “open communication,” and “no secrecy,” and several shared insights into the value placed on openness, such as, “It felt better knowing that we weren’t keeping the secret.” (Parent 55). Parent 44 stated, “I want it to be clear. I want it to be transparent. I don’t want there to be any shame attached to it.” Another reason was fueled by the desire for the child to know they were wanted. Parent 38 said, “I want them to know how wanted they were,” and Parent 32 added, “I think, like I want her to know that I was like really intentional creating her, and that like she’s really valued and loved and appreciated.”

Parents expressed other reasons for needing education and information about how to share their children’s conceptional origins with them. One reason was parents’ perceptions about inadequate education or an alternative focus on the counseling sessions that took place as part of their clinical experience. Parents made statements such as: “We had to talk to someone to get cleared.” (Parent 38), or that they underwent “An evaluation of my husband and me” (Parent 23).

Parents recalled that clinicians provided beneficial education and counseling about the details needed to talk with and share information with their child.

*Part of our fertility clinics requirements in order to do donor egg IVF, was a meeting with a psychiatrist in order to do an evaluation of my husband and me, and I found that evaluation to be really helpful.*

[Parent 58]

However, some parents perceived that no clinician had discussed disclosing the conceptual origins to their children with them. One pregnant parent (21) replied when asked about any clinician addressing disclosure as part of the health care she received, said: “No, not a soul. Nobody except for the therapist, and that was because [it was required].” Another parent (32) shared, “…and no one, no healthcare professional, has talked with me about that at all.”

The clinicians expressed reasons why a tool like the TELL Tool was not only to aid parents in their openness to their children, but they also recognized the often time-limited situations that occur in the clinical setting and the priorities surrounding clinical health care during the patient-clinician encounter.

*In my current practice I am* [time] *limited. We only have 15-minute appointments. And I have to make sure I touch on all the important things about whether they’re getting their third trimester labs on. Or, you know, do they want the new RSV vaccine; and giving them information about that. And they have to check with their insurance company, etc., etc. So, I hadn’t even thought of talking to [patients] about that [disclosure to their children]*.

[Clinician 541]

Clinicians also reported their support for the TELL Tool because of the time spent addressing patients’ emotional needs and the resulting stigma. For example, Clinician 504 said: “So usually what takes the most amount of time is just them telling me about the family situation and how they’re afraid their children [will] get disowned [emphasis added].”

#### Theme 2: Managing Emotions, Conflicts, and Needs

Parents reported a myriad of emotions, including conflict entwined in the processing of using donor-assisted conception and contemplating their future disclosure with their children; and for many, the anticipation of unknown outcomes following telling. Clinicians also recognized parents’ emotional and other needs, as well as their own needs as clinicians.

Most parents expressed the need for emotional support during their clinical care or when transitioning to parenthood. As Parent 55 stated, “I think there was maybe the lack of kind of explaining… the emotional aspect of it…. I think it was just left on our shoulders to try to figure that out.” This notion was also recognized from a clinical standpoint by Clinician 513, as they expressed the need for the tool to “Talk about dealing with the emotional piece.”

Parents went on to report specific emotions, such as experiencing grief, loss, and even trauma that surrounds the use of donor-assisted conception. “It is like death. It is like processing a death.” (Parent 21). Another parent (51) stated, “I did want to process the grief. I wanted to make sure that when I went through this process that I like I had acknowledged [it].” Drawing from clinical experience, clinician 515 supported these sentiments by adding, “So I think it’s a lot of the grief.” Parent 21, who was pregnant at the time of the interview, reflected further about the impact of grief on her ability to tell her future child:

*It’s really emotional going through the process of looking at people’s pictures* [to select a gamete donor]. *It’s like you’re shopping for somebody that’s like you. But nobody is gonna be like you. And you have this weird mixture of excitement but sadness…. And you need to get through all that in order to make it to the end stage of being the parent, and then from there on out, being able to tell them… So that you tell them in a healthy way. And in order to get to the point of being able to tell them in a positive way, you have to process your own grief and trauma*.

Fear of their child rejecting them as a parent when they are told about their donor-assisted conception was acknowledged by many parents. Parents alluded to the emotional pain and the fears that disclosure evokes: “I worry about that hurting my heart” (Parent 53) and “I’m scared to have those conversations” (Parent 38). Parents specifically referred to fearing rejection and hoping for acceptance, as relayed by Parent 36, “…and the biggest question that comes to my head, and my greatest fear is still acceptance. Her, you know, accepting what I did. And not finding faults in it at the end of the day is like my greatest fear.” Parents also voiced concerns about their (future) child navigating feelings and emotions such as shame or resentment, or feelings that would arise from being a “second choice” child. As Parent 49 explained, “I don’t want them to ever feel like they are second choice, because obviously it’s like ‘Oh! She tried, she tried, she tried it, didn’t work, and she couldn’t have one the other way. So her last resort was us.’”

Notably, the emotions parents discussed were not always negative emotions. Rather, parents provided examples of pleasurable emotions such as feeling happy, celebratory, or optimistic. For example, Parent 38 framed her concern about her child not being a “first choice” as a positive emotion. She explained:

*We’re so happy that this is what this is who our family is, and I would want them to understand that piece. I don’t want them to ever feel like that is like something shameful about them, or something that they need to hide or not talk about. I think it’s like it is something that’s slightly different about them than other kids. But like that’s everyone has things that are different about them. And so like, that’s the most important thing for me of just like feeling really proud [of] who they are. …and it wasn’t our first choice. That’s the reality, but it it’s the best choice for us*. [emphasis added]

Managing the emotions that arose from partner conflicts about whether to disclose was viewed as a challenge by parents who were either married or living with a partner. Clinicians also recognized this challenge. While most dyadic partners agreed about sharing information early on: “We’ve typically landed on the same page.” “So, we’re both on the same page” and, “We really didn’t run into any big discrepancies.” (Parents 51, 23 and 27, respectively), several had stark differences as relayed by Parent 55:

*I think the most challenging was the different perspectives we had in the beginning. I mean, we were kind of completely at opposite ends of the spectrum. So that was that probably initially, was probably the most difficult in coming to an agreement.*

In awareness of the interpartner dynamics and conflict that many parents face, Clinician 515 added that parents need to: “Recognize that, you know, if they have a partner, their journey is different than their partners journey.”

As parents delved into their experiences with their partners, how and when to share information was also viewed as a source of conflict. Parent 38 stated: “He’s like, ‘Our kids are so little. Why is this a conversation that we’re having right now?’” And further illustrated by Parent 29, “…I think it was just in the beginning, deciding like how often or how to bring it up to her.” Last, one pregnant parent (26) reported having minimal discussion with their partner, in terms of disclosure, “We’ve not had much discussions about that because we’re a little bit confused [about how to tell]. But I think it’s something that we saved for the future.”

Parents expressed the value placed on and the significance of receiving guidance from a health care provider who was knowledgeable and could field questions about navigating disclosure. And while clinicians, especially those practicing outside of a fertility setting, recognized their own need for education about how to counsel parents on best practices for disclosure, this well-informed counsel was expressed as a need by parents. Clinician 518 said: “Oh, I feel completely unqualified to do that [educate parents about disclosure]. I feel like that’s an incredibly personal, like, parent decision. And I don’t think it’s something that I have any training or knowledge and feel confident, giving any advice about at all whatsoever.” Concurrently, Parent 36 shared, “…make sure that whoever is talking to [you], ... passes the information in a way that is not hurtful that way. You know, you get to derive meaning…” This is further illustrated from the parent vantage point in Parent 44’s observation, “…but I feel like every time I’ve hit a healthcare professional with like, ‘she’s donor conceived,’ like they are like deer in headlights, like they don’t know what to do. They don’t know what to say.*”*

#### Theme 3: Desired Content of a Digital Tool

Parents and clinicians gave invaluable feedback as they reflected on what content or materials of a digital tool would be helpful to them and their patients or clients. Parents emphasized the need for information about understanding how selecting a donor impacts the disclosure process. Parent 27 remarked, “But I do think it’s important to, for parents to know what that [donor selection] process is and isn’t, and sort of the difference between open ID and anonymous [donors].” Parent 44 alluded to regret and how, if better informed, she would not have chosen an “anonymous” donor: “I would not have gone -- I would not have gone with an anonymous donor by any means. I would have entered into a different contract than the one that I am currently in.”

Parents sought guidance and information on how to discuss the donor conception with others, such as family and friends. While Parent 42 asked for guidance specifically on how to talk about disclosure with close family members, Parent 58 expanded on this sentiment: “And then also like what would be our game plan for telling people…close family members, friends, strangers.” Parent 51 acknowledged, “I understand that she [her daughter] lives in a world that’s beyond our little family bubble” and then recounted:

*And then I find when I’m you know, talking to family, they don’t have the language around it… like I said, my mother. my own mother said something about like, “Oh, do you know anything about the mother?” And like, I understand, for some people that would have really hurt.*

From a clinical standpoint, clinicians appreciated the challenge faced by parents and recognized the need for resources to aid in their support of parents. Clinician 515 stated, “To tell the people in their circle, then, is to tell the child. So, maybe telling their parent, or telling their in-laws, or telling their sister or their siblings, or whoever it is, touches that child every day. … I find that that’s a little bit challenging for them as well.” Across interviews with parents and clinicians, requests for and support of insights about the disclosure process from other parents who have experienced it were frequent. Parent 21 described the potential efficacious insights that could be gathered from others who are “ahead of my timeline,” she said:

*But those are the people whose insights I could use more from, I guess, because they’ve already been raising children in this circumstance. And so they’re ahead of my timeline. And I wanna like, just sit down and like talk to them forever like about everything they’ve done -- and what they wish they had done differently. What’s worked, what’s not worked, and what is their plan for the next [step].*

Conversely, one parent (25) explained that hearing from others would not be helpful: “…I’ve noticed that I don’t want to use other people’s methods for my own method, because I tend to, then, second, guess myself. And so I’m planning on just instinctively, you know, going by it rather than creating a narrative, creating a strategy.” This parent acknowledged that if she was underestimating the challenges within the disclosure process, it would be nice to know that the TELL Tool was available.

Parents also recommended that the tool contain insights and stories from DCP. Parent 29 said: “Oh, you know, stories from other, other donor conceived families. But stories from donor conceived adults -- and I think that, that would be more helpful.” Concurring, Parent 32 shared, “… I would have really enjoyed and appreciated hearing other people’s [DCP] stories and reflections on what was important to them growing up that would’ve been super helpful.” Clinician 512 added that having a few DCPs talk about their experiences, such as “Here’s what I wish my parents had said, or here’s what I’m glad my parents have said to me” would be beneficial.

Details about the disclosure process, “I wanted to know the best way to do it.” (Parent 51), including when to disclose and the frequency of follow-up conversations, were mentioned by every parent participant. While some parents touched on being told or knowing they should tell their child when they are “young” or “early” in life and “often” as stated by Parent 44*,* “…We obviously need to tell her early and often, is what I’m gathering.” Yet parents, for the most part, were perplexed by what exactly this meant. Sharing in this sentiment, Parent 42 stated, “Then how do you know if you’re talking too much?” Similarly, Parent 25 thought aloud: “And you’re not gonna have these conversations with the kid until they’re at least 3, I guess right?*”* further illustrating parents’ uncertainty about the disclosure process.

Parents also requested guidance about terminology in relation to donor conception. Parents wanted to know what terms to use for gamete donors as well as others within the donor kinships. As examples, Parent 51 mentioned, “I’m still not sure how to talk about the donor herself. Right now, we’re just talking about the fact that it’s a donor.” As parents thought aloud about the disclosure process, they requested age-appropriate, inclusive language and guidance about uncertainties and important aspects of the conversations. Parent 42 stated, “Even talking points that are age appropriate would be helpful.” Parent 40 said, “What are the most critical things to share with her from a developmental standpoint?”

Parents referred to the potentiality of their children being in contact with or knowing the donor and donor siblings. Thoughts about these potential future relationships sparked parents to request guidance about conversing with their children and navigating these relationships. Parent 49 simply said, “How do I do that? That’s something to think about in the future. Do I connect them? Do I not connect them?” Parent 23 echoed this thought, “… another concern I definitely have is just the concept of other siblings out there -- definitely like [to know] how to handle those conversations.” Further illustrating confusion surrounding this facet, Parent 26 remarked:

*… the kind of relationship I’ll establish between my child’s and the donor’s [child(ren)]. So I feel I’m really so confused…Will I treat them as members of extended family, or I treat them as strangers, and all that? So I’m really confused about that.*

#### Theme 4: Recommended Design and Usability Features

Parents and clinicians reflected and thought aloud about the design, including the tool’s usability features and the optimal time for accessing the tool during the pregnancy and new parenthood periods. Regarding the optimal time, almost all parents indicated that early in the donor-assisted process would be most beneficial. Parent 26 said, “I think the appropriate time should be even before the whole process of maybe going for embryo donation or egg donation.” At the same time, many parents recommended multiple time points as illustrated by Parent 58: “It’s important to do it multiple times. I would recommend it at the initial stage.” Clinicians’ opinions varied on the optimal time for administering the tool. Some clinicians felt it was best used in the fertility setting, while others viewed it as best once pregnancy was established. Other clinicians envisioned multiple times for its use, such as Clinician 510, who said:

*I think we would definitely like send it off with them when they graduate after the pregnancy has been established, and they’re going off to their OB, but I really also think there’s a decent population of women who would find this incredibly helpful in their decision-making process [during initial clinical care].*

In terms of other specific features, slightly over half of the parents spoke of wanting the tool to provide a mechanism for connecting with other parents who were navigating the disclosure process. Parent 42 said, “It’s nice to know you’re not alone.” Parents also recommended a chat feature or the inclusion of forums.

Parents and clinicians spoke of the variability of donor-assisted family types (eg, single mom by choice, heterosexual, same-sex), and shared they would find it helpful if the tool had the ability to provide customization based on family type and also donation type (eg, oocyte, sperm, embryo), as well as customization by the donor anonymity type (ie, directed or nondirected). These suggestions centered on ways to tailor the tool, as Parent 51 illustrated, “The ability to customize some of that information or instructions on how to personalize things” and Clinician 547, who said: “I’d say have it divided into sections like sperm donor conceptions.”

Usability was a key design feature that parents and clinicians recognized as important, and remarked that the difficulty of use could be a challenge to parents. Suggestions included “ease of navigability” (Parent 58) and that the tool be “pretty smooth” and not “clunky” or “wonky” (Parent 21), and in general well-organized. Clinician 533 stated: “It should be easy to use.” Parent 32 summarized these sentiments and said:

*Sometimes the apps can be not very easy to navigate, and my frustration tolerance is really low right now. So, if it isn’t easy or intuitive, if I can’t skim it and kind of see that I’m gonna get something useful out of it, if it’s hard to like get back to where I was. Like, say, the app closes or I get pulled away from my phone… I can imagine that it would be harder for me to use or for me to get much out of it.*

Additionally, parents described how they would ideally engage with a digital tool and their preferences for how the content is delivered. Parents and clinicians pointed out that the tool should be appealing to different types of learners and, as such, would have audio, video, and written text components. Clinician 533 said, “You know some people are auditory learners, you could, you know, have some little clips of clinicians or something, and explaining the process.” In another example, Parent 36 preferred to watch videos and listen to podcasts or audio clips because the tone and emotion helped to make the information clear and provided comfort.

A potential challenge that some parents and clinicians identified with the design was regarding accessibility, particularly the financial cost of accessing the tool. Cost was perceived as a barrier to use, especially if there was a fee associated with its use. Clinician 554 stated: “My patients… are already spending a million dollars trying to do this [donor-assisted conception].*”*

Both parents and clinicians recognized privacy as a potential challenge of using a digital tool. Clinician 518 said, “I think confidentiality could be an issue with them [parents].” Confirming these clinician observations, one parent (26) explained, “I’m much concerned about the data privacy… such that every person’s, every user, every user’s information is protected, and no one else gets to access it.*”* However, another parent (42) stated that although they were not specifically worried about privacy, their partner would see that as a challenge.

Evidence-based information and access to research and articles were revealed as important design features by parents and seen as a requirement of clinicians for a digital tool. Parent 23 stated: “Resources to like studies, maybe, and resources to books that we could -- we could leverage just for us to mentally understand it [talking with their children]” and Parent 32 simply added, “Yeah. Researched information.*”* While Parent 44 shared, “I think it would be huge to have people that are pursuing this path like, to let them know it is actually best for the child, according to most research.” From a clinical standpoint, safety and validity were of utmost importance, as concisely put by Clinician 533: “Has been tested” and illustrated by Clinician 547, “That it’s evidence based.*”*

## Discussion

### Principal Findings

To our knowledge, this study is the first to detail thick, rich data that reports pregnant, expecting, and new parents’ and the clinicians’ perspectives on disclosure to provide a foundation for expanding a digital tool, the TELL Tool, for use during the pregnant and early parenthood periods in the United States. Although we completed an earlier alpha test [32] and a pilot randomized controlled trial [33] using the existing TELL Tool, pregnant women and parents with infants were not eligible for these studies. Thus, from these parents’ and clinicians’ views, 4 themes: “Reasons for What Matters Most;” “Managing Emotions, Conflicts, and Needs;” “Desired Content of a Digital Tool;” and “Recommended Design and Usability Features” were identified that provide detailed and nuanced insight for expanding the TELL Tool into the pregnant and early parenthood periods. For example, the analysis for Theme 1, “Reasons for What Matters Most,” revealed that all 20 parents wanted and intended to inform their children about their donor-conception origins. Their reasons for disclosing varied from wanting to prevent shame and avoiding the harm of secrecy within the family to ensuring their children felt loved and wanted. A 2024 comprehensive review reported similar parental reasons for disclosure [51]. However, we are less certain if all 20 parents will disclose as they intend, as research in the United States has demonstrated that many parents are unable to disclose or have not disclosed even when they have intentions to do so during pregnancy or in early parenthood [52,53]. Nevertheless, knowledge about deeply held reasons for disclosure during pregnancy/early parenthood is important to accomplishing our aim regarding expanding the TELL Tool.

An important finding was the varied experiences reported by parents regarding the Practice Committee of the ASRM’s [2] recommended counseling session for individuals and couples undergoing donor-assisted conception. We found that some parents reported the session as beneficial, while others viewed it as a required step in the process or stated they did not recall any discussion about disclosure with a health care professional. This finding is noteworthy as our sample comprised those who recently underwent treatment (ie, pregnant/new parents) and were closer to recalling a counseling session versus those in our alpha test and pilot randomized controlled trial studies. Although it was beyond the aim of the study to verify if counseling sessions occurred and the quality of the sessions, this finding demonstrates the need for tools to supplement clinical counseling even during pregnancy. The topic of disclosure is one that parents will probably need to hear about multiple times from multiple people over a period of time—in ways that are developmentally appropriate for their children and for them as parents. The TELL Tool provides a detailed way to hear about this information, without the time pressure that office visits entail, and without any knowledge limitations of a particular provider.

This finding also links to the clinicians’ self-identified needs for clinical education and counseling regarding disclosure in Theme 2: “Managing Emotions, Conflicts, and Needs,” especially among those practicing outside of fertility settings. In our prior research, PNPs also expressed this need [25]. We acknowledge the multitude of ethical issues and clinical challenges this quandary presents. Steps such as ASRM’s formation of the “Needs and Interests of Donor Conceived People and Their Families” Taskforce to examine provider education regarding the needs of families formed from gamete donation [54] and European Society of Human Reproduction and Embryology’s practice recommendations [27] and research agenda to include developing innovative care models [55] provide blueprints for needed change. In our own work and given these findings, the next step in our program of research is developing interventions that provide high-quality education for clinicians about disclosure.

Regarding Theme 2*,* parents expressed the challenges of navigating negative (eg, fear, worry) emotions while at the same time appreciating positive (eg, happiness, pride) emotions. These findings imply that counseling via the TELL Tool, or when administered by clinicians, should address emotional support and include aspects of both positive and negative emotional states. Parents also interjected their concerns about their resulting child’s emotional reactions and “acceptance” of their reproductive decision to use donor-assisted conception. Current literature supports early parental disclosure to promote acceptance [19-23] and, more globally, parenting interventions delivered in the first 3 years of a child’s life have been found to support children’s socioemotional development and infant-caregiver attachment in early childhood [56].

Conflict between partners in 2-parent families about disclosure was a concern expressed by both parents and clinicians. The findings highlighted the underlying reasons for dyadic-partner conflict, such as parents’ lack of knowledge and, hence, the disagreement about knowing when to begin telling conversations with their children. This is a key finding that will be an important component for expanding the TELL Tool to this earlier developmental stage of pregnancy/parenting so that parents in 2-parent families can have more time to work out their understandings and reach, ideally, a shared approach.

Participants provided rich contextual information that captured nuances and subtleties for content surrounding disclosure in Theme 3, “Desired Content of a Digital Tool.” Content about how the donor anonymity type (ie, directed, nondirected) can impact disclosure was suggested as helpful information within a digital tool. How to talk with others and learn from others’ disclosure experiences—from both the parent and the DCP perspective—was also touted as essential content. Participants also suggested content on the “best way” or best practices to disclose, such as what age to inform their children and how often to return to disclosure conversations as their child grows. These findings point to parent and clinician recommendations for fostering a “healthy disclosure” that promotes parent, child, and family well-being, which is the fundamental aim of the TELL Tool intervention.

Despite the growing support for openness, understanding the specific components of a healthy disclosure is understudied in the field and provides a critical direction for future research. Notably, evidence supporting early disclosure has burgeoned [19,23] yet gaps remain about other disclosure components that lead to positive or healthy outcomes for parents and children over the life course [55]. For example, Best et al [57] in their reporting of in-depth research with 10 DCPs found that although early disclosure was preferred, other efforts to reduce DCPs’ experiences of stigma and discomfort were the need for ongoing conversations with their parents about their donor-assisted conception. Future research that identifies disclosure components for best practices, such as those identified by Best et al [57] and in this study (eg, consider donor anonymity type when selecting a donor), would be helpful to families and desired content for the expanded TELL Tool.

Another critical need recognized by participants was the recommendation for content about navigating issues regarding the larger donor kinship. Parents and clinicians relayed concerns about talking about kinships and potentially establishing and navigating the presumed complex relationships with the donors, the donor’s family, and donor half-siblings or donor whole-siblings (in the case of embryo donation). Other investigators including ourselves have examined these relationship nuances [19,58-63], and it has been documented that there is an ongoing need for supporting parents in navigating these relationships [64]. Our findings add to the growing call from clinicians, scientists, and parents alike that guidance and clear recommendations are needed to aid families in navigating relationships within the wider donor kinship.

Findings from Theme 4, “Recommended Design and Usability Features,” represent participants’ recommendations for how the tool’s design can best help parents. The findings suggest that information about disclosure is needed early in the donor-assisted conception process. While several parents and clinicians suggested the best timing would be once pregnancy is established, others reported a clear need for information earlier in their clinical care, as gamete donors were being selected. This finding is important as we move forward with implementing design features into the expanded TELL Tool.

The finding that parents requested a tool that would allow them to connect with other parents was also conveyed in our earlier alpha testing research with donor-recipient parents [32]. These parallel findings may be because many parents who use donor-assisted conception constitute a hidden and often stigmatized population, and they are seeking a safe community where they can share similar life experiences. In a related population, and adding support for our interpretation of this finding, Rhee and Kim [65] found that mothers who experienced postpartum depression joined online support groups to fulfill a desire for connectedness and reassurance and to obtain information. Yet, it is possible the need to connect was more prevalent in this select group of parents who volunteered to participate in the study than in the broader population.

Other important findings were the requirement that parents’ privacy be maintained if they use the tool and that it be accessible in terms of cost (ie, minimal cost or free). Given the historical context of donor-assisted conception and the secrecy, stigma, and lack of consistent parentage rights associated with third-party reproduction, we were not surprised that parents viewed privacy as a key feature for the tool. Other investigators have also identified privacy as a key feature of high-quality digital tools designed for parents [66] and that no- or low-cost digital tools increase parents’ accessibility and use [67]. Clinicians voiced an added usability requirement that the tool be validated and found to be effective. In our study with PNPs about implementing the existing TELL Tool into pediatric settings, PNPs emphasized the need for a valid and reliable tool [25], and clinician views in this study were consistent, in further support of those findings. Our next step to further refine the tool is to include testing the TELL Tool tailored to this additional developmental period of pregnancy/early parenthood, as we did with the prior version of the TELL Tool, further strengthening the intervention. Future global research is needed with parents from other countries and from other cultural and political backgrounds to significantly expand knowledge about the disclosure needs of all parents.

### Strengths and Limitations

The primary strength of the study is the engagement of pregnant and new parents who will be the end users of the expanded TELL Tool and the inclusion of clinician perspectives. Involving end users in formative research for developing decision support tools can lead to higher quality and potentially increase usability of the tool [43,45]. Another strength is the thick, nuanced, and insightful data provided by participants about the content and design features for expanding the TELL Tool. Additionally, parent participants were comprised of a range of family types and donor anonymity types, which provides a diverse set of perspectives. In the same vein, clinician experiences in providing health care ranged from 3‐47 years, providing fresh outlooks as well as more seasoned expert perspectives. The clinician’s areas of practice and discipline were varied, providing a breadth of experiences from which to draw insight.

Despite these strengths, the study has several limitations. First, the sample of parents was likely biased toward openness and disclosure by their willingness to participate in the study. We have found in our qualitative [52] and quantitative [33] research that US parents who are less open to disclosure or are more fearful of the consequences of it opt not to participate in our studies examining disclosure. Furthermore, in one of the few longitudinal studies examining disclosure, we found that even though 86% of pregnant women in the sample intended to tell their children or were undecided about whether to tell, only 14% were able to do so at the 12-year follow-up [52]. It is likely that some parents in this study may find disclosure difficult or challenging in the future. The findings need to be viewed through the lens of this sampling bias, such that parents who find disclosure difficult or are considering secrecy during the pregnant and early parenting periods may have other views that were not captured. We acknowledge that parents were English-speaking, US-based, and mostly White and female, which limits transferability, especially to parents of other racial or ethnic backgrounds and those living outside of the United States, where disclosure laws differ, or those who speak other languages. Because the purpose of the study was to expand the TELL Tool for use in the United States, we caution application of the findings to a wider, global audience of parents and clinicians.

### Conclusions

The 4 interrelated themes that were identified from the parents’ and clinicians’ perspectives illustrate the distinct needs and desires of end users and clinicians to provide a formative road map for expanding the TELL Tool for use during the pregnant and early parenthood periods. These insights include knowledge about what drives parents’ decisions for disclosure, which is critical to formulating the expanded content within the TELL Tool. Participants clearly described the impact and subtleties regarding emotions and the need to manage them when planning for and sharing information with children. Understanding nuances, such as between-partner conflicts about disclosure and the need for clinical education, was also an important finding. Thorough and comprehensive information about design and usability features, such as the need for the tool to ensure privacy and be low- or cost-free, was conveyed. Several important areas for future research were also identified. Notably, research that illuminates best practices that can aid parents in navigating the larger donor kinship relationships that disclosure brings forth is needed, as is research that identifies specific disclosure components that can lead to healthy parent and child outcomes over the life course. Future global research among parents from different racial, cultural, and political backgrounds would enhance understanding and likely improve parent and child health as well.

## Abbreviations

ASRM: American Society for Reproductive Medicine
DCP: donor-conceived people
IVF: in vitro fertilization
OB: obstetrician
PNP: pediatric nurse practitioner
RADaR: rigorous and accelerated data reduction technique
RSV: respiratory syncytial virus
TELL Tool: Tool to Empower Parental Telling and Talking

## References

[1] (2025). Using donated eggs, sperm or embryos in treatment. Human Fertilisation and Embryology Authority.

[2] The Practice Committee of the American Society for Reproductive Medicine and the Practice Committee for the Society for Assisted Reproductive Technology (2024). Gamete and embryo donation guidance. Fertil Steril.

[3] De Geyter C, Wyns C, Calhaz-Jorge C (2020). 20 years of the European IVF-monitoring consortium registry: what have we learned? a comparison with registries from two other regions. Hum Reprod.

[4] Croydon S (2023). Reluctant rulers: policy, politics, and assisted reproduction technology in Japan. Camb Q Healthc Ethics.

[5] Salama M, Isachenko V, Isachenko E (2018). Cross border reproductive care (CBRC): a growing global phenomenon with multidimensional implications (a systematic and critical review). J Assist Reprod Genet.

[6] Kupka MS, Chambers GM, Dyer S (2024). International committee for monitoring assisted reproductive technology world report: assisted reproductive technology, 2015 and 2016. Fertil Steril.

[7] Diego D, Medline A, Shandley LM, Kawwass JF, Hipp HS (2022). Donor sperm recipients: fertility treatments, trends, and pregnancy outcomes. J Assist Reprod Genet.

[8] Lee JC, DeSantis CE, Boulet SL, Kawwass JF (2023). Embryo donation: national trends and outcomes, 2004-2019. Am J Obstet Gynecol.

[9] Salazar A, Diaz-García C, García-Velasco JA (2023). Third-party reproduction: a treatment that grows with societal changes. Fertil Steril.

[10] Ombelet W, Van Robays J (2015). Artificial insemination history: hurdles and milestones. Facts Views Vis Obgyn.

[11] Brewaeys A (1996). Donor insemination, the impact on family and child development. J Psychosom Obstet Gynaecol.

[12] Shenfield E (1997). Privacy versus disclosure in gamete donation: a clash of interest, of duties, or an exercise in responsibility?. J Assist Reprod Genet.

[13] Nachtigall RD (1993). Secrecy: an unresolved issue in the practice of donor insemination. Am J Obstet Gynecol.

[14] Daniels KR, Taylor K (1993). Secrecy and openness in donor insemination. Politics Life Sci.

[15] Harper JC, Kennett D, Reisel D (2016). The end of donor anonymity: how genetic testing is likely to drive anonymous gamete donation out of business. Hum Reprod.

[16] Ishii T, de Miguel Beriain I (2022). Shifting to a model of donor conception that entails a communication agreement among the parents, donor, and offspring. BMC Med Ethics.

[17] Calhaz-Jorge C, De Geyter CH, Kupka MS (2020). Survey on ART and IUI: legislation, regulation, funding and registries in European countries: the European IVF-monitoring consortium (EIM) for the European society of human reproduction and embryology (ESHRE). Hum Reprod Open.

[18] Talbot C, Hodson N, Rose J, Bewley S (2024). Comparing the psychological outcomes of donor and non-donor conceived people: a systematic review. BJOG.

[19] Lampic C, Skoog Svanberg A, Gudmundsson J (2022). National survey of donor-conceived individuals who requested information about their sperm donor-experiences from 17 years of identity releases in Sweden. Hum Reprod.

[20] Ilioi E, Blake L, Jadva V, Roman G, Golombok S (2017). The role of age of disclosure of biological origins in the psychological wellbeing of adolescents conceived by reproductive donation: a longitudinal study from age 1 to age 14. J Child Psychol Psychiatry.

[21] Jadva V, Freeman T, Kramer W, Golombok S (2009). The experiences of adolescents and adults conceived by sperm donation: comparisons by age of disclosure and family type. Hum Reprod.

[22] Quintigliano M, Carone N, Speranza AM (2022). Adolescent development and the parent-adolescent relationship in diverse family forms created by assisted reproduction. Int J Environ Res Public Health.

[23] Golombok S, Jones C, Hall P, Foley S, Imrie S, Jadva V (2023). A longitudinal study of families formed through third-party assisted reproduction: mother-child relationships and child adjustment from infancy to adulthood. Dev Psychol.

[24] Boyle WE, McInerny TK, Adam HM, Campbell DE (2017). American Academy of Pediatrics Textbook of Pediatric Care.

[25] Hershberger PE, Adlam K, Driessnack M (2025). Parent and pediatric nurse practitioner views on integrating the digital TELL tool intervention into clinical practice. J Pediatr Nurs.

[26] Ethics Committee of the American Society for Reproductive Medicine. Electronic address: ASRM@asrm.org, Ethics Committee of the American Society for Reproductive Medicine (2018). Informing offspring of their conception by gamete or embryo donation: an ethics committee opinion. Fertil Steril.

[27] Kirkman-Brown J, Calhaz-Jorge C, ESHRE Working Group on Reproductive Donation (2022). Good practice recommendations for information provision for those involved in reproductive donation^†^. Hum Reprod Open.

[28] Hershberger PE, Gallo AM, Adlam K (2023). Development of the tool to empower parental telling and talking (TELL Tool): a digital decision aid intervention about children’s origins from donated gametes or embryos. Digit Health.

[29] Piaget J, Inhelder B (1969). The Psychology of the Child.

[30] Scott HK, Piaget CM (2022). StatPearls.

[31] Wadsworth BJ (2004). Piaget’s Theory of Cognitive and Affective Development.

[32] Hershberger PE, Gallo AM, Adlam K (2022). Alpha test of the donor conception tool to empower parental telling and talking. J Obstet Gynecol Neonatal Nurs.

[33] Hershberger PE, Gruss V, Steffen AD (2025). A randomized pilot trial of the donor conception tool to empower parental telling and talking (TELL Tool) with their children about their genetic origins. J Pediatr Health Care.

[34] Santaguida E, Bergamasco M (2024). A perspective-based analysis of attachment from prenatal period to second year postnatal life. Front Psychol.

[35] Glover V, Capron L (2017). Prenatal parenting. Curr Opin Psychol.

[36] Watkins DC (2017). Rapid and rigorous qualitative data analysis: the “radar” technique for applied research. Int J Qual Methods.

[37] Lewinski AA, Crowley MJ, Miller C (2021). Applied rapid qualitative analysis to develop a contextually appropriate intervention and increase the likelihood of uptake. Med Care.

[38] Bradshaw C, Atkinson S, Doody O (2017). Employing a qualitative description approach in health care research. Glob Qual Nurs Res.

[39] Sandelowski M (2010). What’s in a name? qualitative description revisited. Res Nurs Health.

[40] Patton MQ (2015). Module 38: Mixed, Stratified, and Nested Purposeful Sampling Strategies.

[41] Sepucha KR, Abhyankar P, Hoffman AS (2018). Standards for universal reporting of patient decision aid evaluation studies: the development of SUNDAE checklist. BMJ Qual Saf.

[42] Hoffman AS, Sepucha KR, Abhyankar P (2018). Explanation and elaboration of the standards for universal reporting of patient decision aid evaluations (SUNDAE) guidelines: examples of reporting SUNDAE items from patient decision aid evaluation literature. BMJ Qual Saf.

[43] Witteman HO, Maki KG, Vaisson G (2021). Systematic development of patient decision aids: an update from the IPDAS collaboration. Med Decis Making.

[44] Elwyn G, Kreuwel I, Durand MA (2011). How to develop web-based decision support interventions for patients: a process map. Patient Educ Couns.

[45] Coulter A, Stilwell D, Kryworuchko J, Mullen PD, Ng CJ, van der Weijden T (2013). A systematic development process for patient decision aids. BMC Med Inform Decis Mak.

[46] Patton MQ (2015). Designing Qualitative Studies Qualitative Research & Evaluation Methods.

[47] Hershberger PE, Gallo AM, Adlam K (2021). Parents’ experiences telling children conceived by gamete and embryo donation about their genetic origins. F S Rep.

[48] Eccles DW, Arsal G (2017). The think aloud method: what is it and how do I use it?. Qual Res Sport Exerc Health.

[49] Wolcott MD, Lobczowski NG (2021). Using cognitive interviews and think-aloud protocols to understand thought processes. Curr Pharm Teach Learn.

[50] Noushad B, Van Gerven PWM, de Bruin ABH (2024). Twelve tips for applying the think-aloud method to capture cognitive processes. Med Teach.

[51] Duff MA, Goedeke S (2024). Parents’ disclosure to their donor-conceived children in the last 10 years and factors affecting disclosure: a narrative review. Hum Reprod Update.

[52] Hershberger PE, Driessnack M, Kavanaugh K, Klock SC (2021). Oocyte donation disclosure decisions: a longitudinal follow-up at middle childhood. Hum Fertil (Camb).

[53] Applegarth LD, Kaufman NL, Josephs-Sohan M, Christos PJ, Rosenwaks Z (2016). Parental disclosure to offspring created with oocyte donation: intentions versus reality. Hum Reprod.

[54] American Society for Reproductive Medicine (2022). ASRM announces taskforce on the interests of donor conceived people. Press Release Point.

[55] Sermon K, Coticchio G, Delbaere I, Fadler C, Gameiro S, European Society of Human Reproduction and Embryology (ESHRE) Working Group on Research Gaps and Priorities (2025). Impacting infertility: a research agenda. March 2025.

[56] Jeong J, Franchett EE, Ramos de Oliveira CV, Rehmani K, Yousafzai AK (2021). Parenting interventions to promote early child development in the first three years of life: a global systematic review and meta-analysis. PLoS Med.

[57] Best S, Goedeke S, Thorpe M (2023). Make our wellbeing a priority: donor-conceived adults call for ongoing support and conversation about their donor conception. Hum Fertil (Camb).

[58] Crawshaw M, Daniels K, Adams D (2015). Emerging models for facilitating contact between people genetically related through donor conception: a preliminary analysis and discussion. Reprod Biomed Soc Online.

[59] Scheib JE, McCormick E, Benward J, Ruby A (2020). Finding people like me: contact among young adults who share an open-identity sperm donor. Hum Reprod Open.

[60] Zadeh S, Ilioi EC, Jadva V, Golombok S (2018). The perspectives of adolescents conceived using surrogacy, egg or sperm donation. Hum Reprod.

[61] Hertz R, Nelson MK, Kramer W (2017). Donor sibling networks as a vehicle for expanding kinship: a replication and extension. J Fam Issues.

[62] Koh AS, van Beusekom G, Gartrell NK, Bos H (2020). Adult offspring of lesbian parents: how do they relate to their sperm donors?. Fertil Steril.

[63] Hershberger PE, Driessnack M, Kavanaugh K, Klock SC (2020). Emerging views of kinships created through oocyte donation. MCN Am J Matern Child Nurs.

[64] Schrijvers AM, van Rooij FB, de Reus E (2020). Psychosocial counselling in donor sperm treatment: unmet needs and mental health among heterosexual, lesbian and single women. Reprod Biomed Online.

[65] Rhee ES, Kim HS (2023). Understanding the dynamics of online social support among postpartum mothers in online communities. Matern Child Health J.

[66] Virani A, Duffett-Leger L, Letourneau N (2019). Parenting apps review: in search of good quality apps. Mhealth.

[67] Doty JL, Brady SS, Monardez Popelka J (2020). Designing a mobile app to enhance parenting skills of Latinx parents: a community-based participatory approach. JMIR Form Res.

